# Accuracy and Reliability of Intermittent Scanning and Real-Time Continuous Glucose Monitoring Systems in Diabetes Emergencies

**DOI:** 10.1177/19322968251334633

**Published:** 2025-04-23

**Authors:** Lukas van Baal, Lutz Heinemann, Johanna Reinold, Jill von Conta, Fin Hendrik Bahnsen, Jens Kleesiek, Dagmar Fuehrer, Susanne Tan

**Affiliations:** 1 Department of Endocrinology, Diabetes and Metabolism, University Hospital Essen, University of Duisburg-Essen, Essen, Germany; 2 Science Consulting in Diabetes, Düsseldorf, Germany; 3 Department of Nephrology, University Hospital Essen, University of Duisburg-Essen, Essen, Germany; 4 Institute for Artificial Intelligence in Medicine, University Hospital Essen, University of Duisburg-Essen, Essen, Germany

**Keywords:** accuracy, continuous glucose monitoring (CGM), hospital, hyperglycemia, hypoglycemia, inpatient

## Abstract

**Background:**

Diabetes care is a major challenge of patients treated in hospitals. A continuous glucose monitoring system (CGM) provides a more comprehensive assessment of glucose control than capillary blood glucose measurements. Especially in emergencies, data on CGM use in inpatients are limited. To evaluate real-world usability, accuracy of an intermittent scanning and a real-time CGM in patients admitted due to diabetes emergencies was assessed.

**Methods:**

In 151 patients admitted due to diabetes emergencies, this single-center prospective study investigated the mean absolute relative difference (MARD) in broad glycemic ranges. The CGM accuracy was evaluated by applying a modified version of the Food and Drug Administration (FDA) criteria for CGM use, Clark Error Grid (CEG), and Bland Altman analysis (BAA).

**Results:**

Analysis of 1,498 CGM-/POC-glucose (CGM-/POC-G) pairs revealed a MARD of 10.8% with stepwise improvement from the hypoglycemic to the hyperglycemic range. The CEG analysis showed that 99.1% of all glucose values fell within the optimal or acceptable zones. BAA indicated that 96.0% of CGM-G values fell within the limits of the POC-G values. Day-by-day analysis of overall MARD revealed the highest MARD on the first day of CGM use, followed by consistent and stable MARD levels from day 2 onward until the end of wear time. Applying a modified version of the %20/20 agreement rate of the FDA criteria, 90.7% of CGM-G laid within ±20 mg/dl/±20% agreement rule.

**Conclusion:**

This study indicates the usability of CGM for inpatient diabetes care by demonstrating a high accuracy and reliability of CGM measurement.

## Introduction

Diabetes as one of the most common in-hospital comorbidities is associated with an increased risk of complications, length of stay in the hospital, and higher mortality.^1–3^ Achieving safe and effective glucose control in inpatients presents a significant and hitherto inadequately addressed challenge.^4,5^ Point-of-care capillary blood glucose (POC-G) measurements to guide diabetes therapy of hospitalized patients is time-consuming for both patients and medical staff offering only limited information about the glucose course with in average four glucose values per day.^6^ In consequence, one-third of postprandial hyperglycemic events and up to 90% of hypoglycemic events among hospitalized patients with diabetes remain undetected.^7,8^ A continuous glucose monitoring system (CGM) provides a more comprehensive assessment of glucose levels and may offer valuable insights that help to guide insulin therapy potentially improving in-hospital diabetes with reduced diabetes management burden and an increase inpatients’ safety.^9^ Due to demonstrated advantages of CGM- over POC-G testing in the outpatient setting guidelines recommend for continued use of CGM in those already equipped with a CGM before hospitalization.^10,11^ Moreover, in-hospital initiation of CGM is recommended in patients with diabetes and high risk for hypoglycemia.^12^ However, to implement CGM into hospital care, information on accuracy and reliability is needed. Number of studies on the performance of CGM in the hospital are limited and performed mostly in size-limited patient cohorts. The first studies revealed mediocre and variable CGM accuracy with a mean absolute relative difference (MARD) ranging between 12.9% and 21.4%.^13,14^ Since CGM technology is rapidly evolving, data on accuracy and reliability are also needed for the latest CGM generations. The aim of this study is to assess the accuracy and reliability of two widely used CGM sensors (FreeStyle Libre 2 (FSL2) and 3 (FSL3); Abbott, Wiesbaden, Germany) in a real-world hospital setting with inpatients admitted due to diabetes emergencies.

## Methods

### Study Design

This single-center prospective study was conducted at the Department for Endocrinology, Diabetology and Metabolism of the University Hospital Essen and included patients admitted from March 1, 2020, to August 31, 2023, to the department’s ward due to hyperglycemic diabetic emergencies. The following situations were classified as hyperglycemic diabetic emergencies requiring inpatient care and continuous intravenous insulin infusion (CIII) (1) mild or moderate diabetic ketoacidosis (DKA; plasma glucose >250 mg/dl and pH 7.25-7.30 or pH 7.0-7.25), (2) acute hyperglycemia defined by random plasma glucose >300 mg/dl, and/or (3) chronic hyperglycemia defined by an HbA1c >9.0%. Patients with severe DKA (plasma glucose >250 mg/dl and pH <7.0) or hyperglycemic hyperosmolar state (HHS; plasma glucose >600 mg/dl) with a Glasgow-Coma-Scale ≤11 at admission were initially monitored and treated at an intensive care unit (ICU) and transferred to our department as soon as a stable state of pH ≥7.0 and or a Glasgow-Coma-Scale >11 were achieved. As part of the university hospital’s quality-improvement project SmartDiabetesCare (QiP SDC), these patients received a CGM at admission on the department’s ward, which measured subcutaneous interstitial fluid glucose concentrations continuously and a smartphone (iPhone SE, Apple, USA) receiving data from the CGM via an application (FreeStyle LibreLink). From March 1, 2020 to June 30, 2020, patients received a FSL2, and from July 1, 2020 to August 31, 2023, a FSL3 sensor. Glucose data transfer was performed by intermittent scanning for FSL2 and in real time for FSL3. Data were shared with the nursing team of the ward from application to application (LibreLinkUp) and cloud-based with the diabetes team (LibreView).

The glucose sensors of the CGM were applied on first day of admittance by a member of the diabetes team and remained placed until the day of discharge or at maximum for 14 days. Whenever the length of hospital stay exceeded wear time, the first sensor was removed and a second was inserted. In addition, the health care teams were instructed to remove the sensor in case of a MRI examination or in case of a local skin reaction, which, however, was not necessary in any patient.

During CIII, nursing staff was asked to document glucose values obtained by CGM once per hour. With multiple daily insulin injections (MDI), CGM-G was documented at least four times per day (before meals and once in the night). At least every six hours (twice per early and midday shift and once per night shift) POC-G measurements with a maximum time lag less than two minutes to CGM measurement were performed with a StatStrip glucose meter (Nova Biomedical, Mörfelden-Walldorff, Germany; coefficient for the slope of a laboratory glucose measurement system (Yellow Spring Instrument) vs. StatStrip glucose meter: 1.023 (r = 0.989) with all StatStrip glucose meter readings within the A zone of Clark Error Grid analysis (CEG).^15^ In defined situations, nursing staff were asked to perform an additional glucose control by POC-G (e.g. if glucose values are not credible to the nursing staff’s opinion). In case of a significant discrepancy between CGM-G and POC-G value, POC-G was used to guide further treatment. Glucose data were documented with corresponding CGM-G data in the patient’s electronic healthcare report (EHR) resulting in at least five CGM-/POC-G pairs per patient and day. Insulin perfusion rates (human insulin; Huminsulin normal^©^, Eli Lilly and Company, Indianapolis, USA) during CIII were adjusted based on the CGM-G data according to the department’s insulin infusion protocol (Supplementary Figure 1). When target glucose values were reached, CIII was switched to MDI with/without oral antidiabetic drugs. Clinical and anthropometric data were extracted from the EHR. EHR of study participants were also checked for substances reported to interfere with the glucose measurement of the CGM (acetaminophen >4 g/d, acetylsalicylic acid >100 mg/d, ascorbic acid >500 mg/d, or a tetracycline).^16^

Informed consent was obtained from all individual participants included in the study. This study was performed in line with the principles of the Declaration of Helsinki. The study has been approved by the ethics committee of the University Hospital Essen (20-9333-BO).

### Statistical Analysis

Relative differences (RD) between glucose values obtained by the CGM and the point-of-care system were calculated as RD = 100 × (CGM-G − POC-G) / POC-G. Non-paired glucose values were excluded from the data analysis. The MARD were calculated for each patient and the patient population. The resulting number of pairs was sufficient to depict medium effect sizes (f = 0.25) with a good statistical power of 0.9.

Categorical variables were compared using chi-squared test, normally distributed variables using independent sample t-test and non-normally distributed variables, such as MARD, using Mann-Whitney U-test. Multiple regression analysis was performed to evaluate for correlation between MARD and age, sex, body mass index (BMI), type of diabetes, hemoglobin, hemoglobin A1c (HbA1c), bilirubin and glomerular filtration rate (GFR).

Outcome measures were (1) MARD between CGM-G and POC-G within the following glucose ranges: <70 mg/dl, 70 to 180 mg/dl, 181 to 250 mg/dl, >250 mg/dl, and combined; (2) the number and percentage of glucose values within ±20% of POC-G ≥70 mg/dl or ±20 mg/dl of POC-G <70 mg/dl, in accordance from the AIDING study, which provide a modified version of the %20/20 agreement rate of the Food and Drug Administration (FDA) criteria for CGM use and is similar to protocols developed by others.^17–19^ For the sake of clarity, the term “%20/20” will be used throughout the rest of the manuscript. CEG and Bland Altman analysis (BAA) were performed as established.^20,21^ All calculations were performed by using the statistical program for social sciences (SPSS) version 28 (IBM, New York, USA). The graphs were generated with GraphPad Prism (GraphPad Software Inc., San Diego, CA, USA). A value of *P* < .05 was considered statistically significant.

## Results

### Patient Characteristics and CGM Data

A total of 151 patients with emergency admittance due to hyperglycemic events were included in the study. In this cohort, 96 had type 2 diabetes (63.6%), 31 type 1 diabetes (20.5%) and 24 other types of diabete, e.g. immune checkpoint inhibitor-induced or post-transplant diabetes (15.9%). The patients had a mean age of 54.3 ± 17.9 years, BMI of 27.6 ± 7.5 kg/m², inpatient plasma glucose of 157 ± 68 mg/dl (minimum: 39 mg/dl; maximum: 517 mg/dl) and HbA1c of 9.9 ± 2.8% (Table 1).

**Table 1.** Demographic Characteristics of the Study Population.

	Total	FSL2	FSL3	*P*-value
N	151	42	109	
Age, years	54.3 ± 17.9	53.3 ± 18.1	54.7 ± 18.0	.34
Female, n (%)	74 (49.0)	20 (47.6)	54 (49.5)	.83
CGM wear time, days	7.8 ± 3.7	8.2 ± 3.7	7.7 ± 3.7	.14
BMI, kg/m^2^	27.6 ± 7.5	28.9 ± 8.2	27.1 ± 7.3	.11
HbA1c, %	9.9 ± 2.8	10.5 ± 3.0	9.4 ± 2.6	.08
Glucose, mg/dl	157 ± 68	153 ± 69	169 ± 72	.22
Glucose at admission, mg/dl	362 ± 72	355 ± 67	364 ± 78	.50
Type 1 Diabetes, n (%)	31 (20.5)	7 (16.7)	24 (22.0)	.47
Type 2 Diabetes, n (%)	96 (63.6)	28 (66.6)	68 (62.4)	.63
Other, n (%)	24 (15.9)	7 (16.7)	17 (17.6)	.90
Hemoglobin, g/l	123 ± 21	124 ± 24	122 ± 20	.60
Mild anemia hemoglobin (100-120 g/l), n (%)	49 (30.4)	12 (28.6)	37 (33.9)	.53
Moderate-severe anemia hemoglobin (<100 g/l), n (%)	21 (13.9)	7 (16.7)	14 (12.8)	.54
Glomerular filtration rate (ml/min/1.73 m^2^)	54.6 ± 17.9	55.3 ± 19.1	54.4 ± 17.5	.78
Chronic kidney disease, n (%)	74 (49.0)	20 (47.6)	54 (49.5)	.84
CKD G1, n (%)	0 (0)	0 (0)	0 (0)	1
CKD G2, n (%)	15(9.9)	4 (9.5)	11 (10.1)	.91
CKD G3a, n (%)	28 (18.5)	5 (11.9)	23 (21.1)	.16
CKD G3b, n (%)	17 (11.3)	8 (19.1)	9 (8.3)	.06
CKD G4, n (%)	8 (5.3)	2 (4.8)	6 (5.5)	.86
CKD G5, n (%)	6 (4.0)	1 (2.4)	5 (4.6)	.54
Charlson score	4.6 ± 4.1	4.5 ± 4.3	4.6 ± 4.0	.89
Bicarbonate at admission, mmol/l	23.2 ± 7.3	23.8 ± 6.8	22.9 ± 7.5	.76
Lactate, mmol/l	1.7 ± 0.7	1.9 ± 0.7	1.7 ± 0.7	.11
Total bilirubin, mg/dl	0.6 ± 0.6	0.7 ± 0.8	0.6 ± 0.5	.36
Acetylsalicylic acid, n (%)	34 (22.5)	11 (26.2)	23 (21.1)	.50
Ascorbic acid, n (%)	0 (0.0)	0 (0.0)	0 (0.0)	1
Acetaminophen, n (%)	2 (1.3)	1 (2.4)	1 (1.0)	.51
Tetracycline, n (%)	0 (0.0)	0 (0.0)	0 (0.0)	1
Hydroxyurea, n (%)	0 (0.0)	0 (0.0)	0 (0.0)	1

Data are presented as mean ± standard deviation or absolute numbers and percentage affected; *P*-value provided for comparison of FSL2 vs. FSL3. FSL2, FreeStyle Libre 2; FSL3, FreeStyle Libre 3; BMI, body mass index; CKD, chronic kidney disease.

At the time of admission, the mean plasma glucose was 362 ± 72 mg/dl with acute hyperglycemia present in 103 patients (68.2%), mild DKA in 21 patients (13.9%), moderate DKA in 13 patients (8.6%) and 14 patients with chronic hyperglycemia (9.3%). Of note, due to the need for intensive care because of severe DKA (7/151, 4.6%), or due to an HHS with a GCS ≤11 (5/151, 3.3%), 12 patients did not receive a CGM at admission but later at the first day on the department’s ward. Mean lactate level was 1.7 ± 0.7 mmol/l, bicarbonate 23.2 ± 7.3 mmol/l, and Charlson Score 4.6 ± 4.1 at admission.

Mean time with CIII was 84 hours, and mean time with MDI 108 hours. Forty-two patients used FSL2 (27.8%), and 109 FSL3 (72.2%). In total, 1498 CGM-/POC-G pairs (521 FSL2, 977 FSL3) were obtained. While all pairs in the normoglycemic and hyperglycemic range were obtained equally during CIII and MDI, all pairs in the hypoglycemic range were observed during MDI. Baseline characteristics did not differ significantly among users of the CGMs studied. Concerning potentially interfering substances, 22.5% of patients were treated with acetylsalicylic acid; however, none received acetylsalicylic acid in doses >100 mg per day. Of the total, 1.3% of patients received acetaminophen with no patient exceeding a daily dose above 4 g. None of the included patients were treated with ascorbic acid, hydroxyurea, or tetracycline.

### Accuracy and Reliability of CGM

Overall MARD of CGM-G for both sensor types was 10.8% and did not differ between the CGMs studied (Table 2). MARD of CGM improved across the glucose range from the hypoglycaemic to the hyperglycemic range, with a MARD <70 mg/dl of 13.9% to 7.7% >250 mg/dl (*P* = .04). No trend for high or low measuring in relation to its paired POC-G was observed (CGM-G > POC-G: 50.5% vs. CGM-G < POC-G: 49.5%, *P* = .58).

**Table 2.** Overall MARD of CGM-G across the glycemic range.

POC-G (mg/dl)	Overall MARD, % (n)	FSL2 MARD, % (n)	FSL3 MARD, % (n)	*P*-value
Overall	10.8 (1498)	11.1 (521)	10.6 (977)	.21
<70	13.9 (65)	22.3 (9)	12.6 (56)	.12
70-179	11.3 (1002)	11.3 (345)	11.3 (657)	.48
180-250	9.7 (188)	10.3 (123)	9.3 (177)	.20
>250	7.7 (131)	8.9 (44)	7.1 (87)	.08

Data are presented as MARD and absolute numbers of CGM-/POC-G pairs; *P*-value provided for MARD comparison of FSL2 vs. FSL3. POC-G, point-of-care glucose; MARD, mean absolute relative difference; FSL2, FreeStyle Libre 2; FSL3, FreeStyle Libre 3; CGM, continuous glucose monitoring system; n, number.

**Figure 1.**
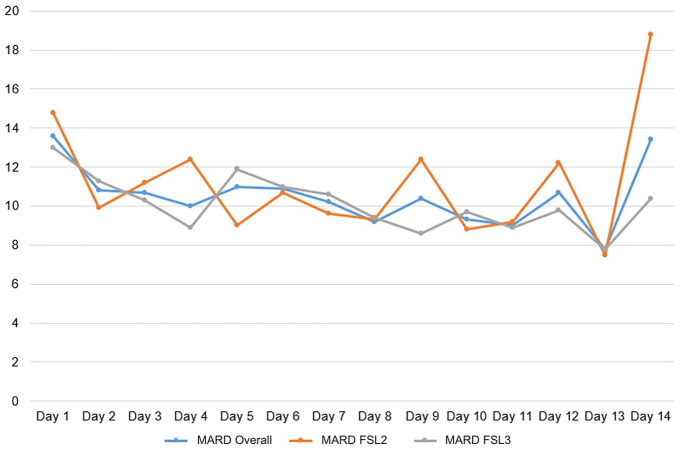
Overall MARD for both sensors combined (blue), and separately FSL2 (orange), and FSL3 (gray) over wear time. MARD, mean absolute relative difference; FSL2, FreeStyle Libre 2; FSL3, FreeStyle Libre 3.

Analysis of day-by-day overall MARD indicated the highest MARD at day 1 of CGM use with constant MARD over time from day 2 on until the last day of system use (day 1 vs. days 2-14: 13.6% vs. 10.4%, *P* < .01; Figure 1). This finding was also observed when data were analyzed separately for the CGMs studied (Figure 1).

**Figure 2.**
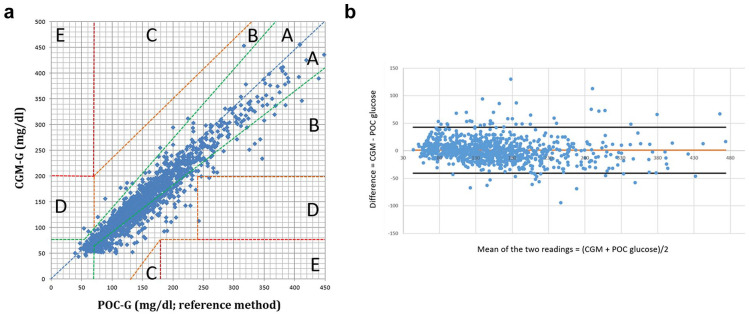
Clarke Error Grid (CEG) and Bland Altman analysis (BAA) for all CGM-/POC-G pairs (n = 1498). (a) CEG for all CGM-/POC-G pairs (n = 1498) within the measurement range of the FSL2 and FSL3; (b) Bland Altman plot of difference (CGM-POC) versus mean. This plot shows the difference between CGM-G and POC-G 1498 matched data pairs vs. their mean. The mean difference is given by the solid line. Dashed lines note 2 standard deviation limits and provide an estimate of where 95% of the differences lie. Abbreviations: CGM-G, continuous glucose monitoring glucose value; POC-G, point-of-care glucose value; CGM, continuous glucose monitoring system; POC, point-of-care system.

The CEG analysis of CGM-/POC-G pairs showed that 99.1% of all CGM-derived glucose values fell within the optimal (zone A) or acceptable (zone B) regions, while 0.9% were in the critical error D and E regions (Figure 2). A comparison of the CEG analysis did not reveal a significant difference between the CGMs studied (*P* = .09, Supplementary Figures 2 and 3). All values in D and E regions occurred in four individuals, with no individual having more than one value in zones D/E. 50% of values in D and E regions occurred within 24 hours after the start of CGM use, and 50% within the last 24 hours of CGM use.

BAA showed that 96.0% (n = 1438/1498) of glucose values fell within the limits of agreement (= mean difference between CGM-G and POC-G ± 1.96 SD of differences), without indicating a systematic error in glucose measurement by either CGM (Figure 2). Twenty-seven of 88 (30.7%) values that were outside the limit of agreements were recorded on the day 1 of system use. Comparison of BAA did not show a significant difference between the results of the CGMs studied (Supplementary Figures 2 and 3).

When applying the %20/20, 90.7% of CGM-G values met these criteria (Table 3). Percentage of CGM-G meeting the %20/20 did not differ significantly between the CGMs studied. Among the glucose data pairs lying outside the %20/20 28.1% (n = 39/139) were observed on day 1. Accordingly, a significantly lower percentage of glucose data pairs fulfilled the %20/20 on day 1 of CGM use compared to day 2 and subsequent days (76.6% vs. 87.7%, *P* < .01; Table 3). From day 2 until day 14, stable day-by-day performance of both CGMs was observed (Supplementary Tables 1 and 2). Multiple regression analysis did not reveal significant results between MARD and analyzed covariates (Supplementary Table 3).

**Table 3.** Accuracy of CGM-G vs. POC-G according to the %20/20.

	Within ±5 mg/dl; ±5%	Within ±10 mg/dl; ±10%	Within ±12 mg/dl; ±12%	Within ±15 mg/dl; ±15%	Within ±20 mg/dl; ±20%	Total no. of pairs
Day 1	34.0%	59.2%	62.9%	68.8%	75.6%	159
Day 2+	33.3%	57.9%	66.8%	77.7%	87.7%	1339
*P*	.86	.75	.33	**.01**	**<.01**	1.498

Data are presented as percentage affected; *P*-value is provided for comparison of percentage affected on day 1 vs. day 2+, bold values are considered statistically significant.

## Discussion

In a cohort of patients admitted to the hospital due to hyperglycemic emergencies, CGM accuracy was 10.8% with a sufficient reliability of two CGMs, including a real-time scanning CGM.

Our study including a real-time CGM in a non-ICU setting showed a MARD of 10.8% in 1498 CGM-/POC-G pairs (FSL2: 11.1%; FSL3: 10.6%). Previous studies in inpatients revealed a MARD of 18% to 21% for the FSL1 and FSL2 in the study by Wright et al. and 14.8% for the professional version of this CGM FSL-Pro in the analysis by Galindo et al.^14,22^ Real-world data provided by Wang et al.^23^ focusing on patients with type 1 diabetes already equipped with a CGM before hospitalization revealed a MARD of 12.3% during hospitalization. Considering a higher MARD on day 1 as demonstrated in our study, the higher MARD in the study of Wright et al.^14^ may be explained by differences in the CGM generations used and by a shorter wear time (2.0 vs 8.0 days). In addition, Wright et al. not only investigated less CGM-/POC-G-pairs (n = 676 vs. 1498) but also allowed higher time lag within the CGM-/POC-G pairs (15 minutes vs. 2 minutes). With a comparable high number of data glucose pairs and a time delay of five minutes between CGM-G and POC-G, Galindo et al. observed a MARD of 14.8%, and Wang et al. of 12.3% which aligns more closely with our study.^22,23^ In addition to the demonstrated high accuracy, we could exclude a systematic error in CGM-based glucose measurement by BAA, underlining that CGM represents a reliable tool for inpatient glucose monitoring.^21^

Besides the overall accuracy, high accuracy of CGM from the hypoglycemic to the hyperglycemic range has to be ensured.^24–26^ The prevalence of hypoglycemic and hyperglycemic events in the studies published hitherto was rather low.^14,27^ By focusing on patients with diabetes emergencies, our study adds knowledge on accuracy in the acute hypoglycemic and severe hyperglycemic range. We showed a MARD of 7.7% in 131 pairs of level 2 hyperglycemia and of 13.9% in 65 pairs within the hypoglycaemic range. For comparison, Wright et al. found a MARD of 19.0% in level 2 hyperglycemia and of 7.6% within the hypoglycaemic range, but with lower pairs.

Furthermore, integrating CGM into routine inpatient diabetes care enables treatment decisions to be based on real-time CGM data. CEG analysis of our CGM-/POC-G pairs revealed minimal risk for inappropriate treatment of hypoglycaemia or hyperglycemia when using the CGMs studied, thus indicating that obtained CGM-G values are reliable for in-hospital insulin dosing. This is in line with CEG analyses of previous studies that investigated the predecessors of the here analyzed CGMs.^14,22^ Moreover, for CGM integration into inpatient care, meeting the %20/20 is required, but studies addressing the %20/20 are limited.^28^ We revealed, that both investigated CGMs performed sufficiently with 90.7% of all CGM-G values fulfilling the %20/20-AR, while Galindo et al. examining the FSL-Pro reported that only 3 out of 4 CGM-G values fulfilled the %20/20.^22^

Another key point highly relevant for the integration of CGM into in-hospital diabetes management is a sustained accuracy during the whole hospital stay. Hitherto this topic was only investigated in one study with another CGM (Dexcom G6). The study showed consistent accuracy over sensor wear time.^29^ However, the median and maximum wear time in this study were short with three and six days, respectively. Therefore, comparability with the real-world setting of inpatient diabetes care is limited. In our real-world study with a median and maximum wear time of 8 and 14 days, respectively, a constant CGM accuracy throughout the hospitalization from day 2 until day 14 of sensor wear time could be shown. The higher MARD observed on day 1 is probably associated with local inflammation after sensor insertion and could also be explained by lower tissue perfusion during the initial phase of severe hyperglycemia.^30,31^ Our findings with a high MARD and optimal chance for meeting the %20/20 from day 2 on underline the usefulness of a hybrid procedure on day 1 but also indicate the option of a less-frequent concomitant POC-G test from day 2 until end of wear time.

### Limitations and Strengths

POC capillary blood glucose rather than laboratory analysis of venous plasma glucose concentrations (Lab-G) was used as reference measurement for the accuracy assessment of CGM-G. However, studies comparing CGM-G with POC-G as well as Lab-G reported comparable MARD values for CGM-/Lab-G and CGM-/POC-G.^32^ Furthermore, POC-G measurement is the standard method for glucose monitoring in clinical practice guiding insulin treatment. Thus, our approach offers direct comparison of CGM with a real-world comparator.^5,33^ It has been demonstrated that acidosis, is associated with impaired CGM accuracy.^34^ Our protocol did not include repetitive measurements of bicarbonate. However, in our non-ICU setting, bicarbonate was measured once at admission with a mean bicarbonate in the lower reference range. It can be assumed that bicarbonate levels do not rise during consecutive reconstitution of acidosis under insulin treatment. Therefore, we do not assume a significant impact of acidosis resp. bicarbonate levels on our results.

Using a well-defined patient cohort with hyperglycemic emergencies and consecutive glucose recompensation, the strength of our study is the high number of CGM-/POC-G data pairs obtained across the entire glycemic range with a low time gap between CGM-G and POC-G and a structured evaluation and documentation of CGM-G and POC-G values. Moreover, we provide an analysis of accuracy over a longer wear time than previously described.

## Conclusions

This study showed a high accuracy of CGM-derived glucose values for patients admitted due to diabetes emergencies. Meeting the FDA requirements, usability of CGM for non-ICU inpatient diabetes emergency care is suggested. Data on clinical outcomes as well as studies on glucose metrics of special interests (e.g. hypoglycemia) and special settings (e.g. perioperative setting) are needed to further evaluate the benefit of CGM in the hospital.

## Supplemental Material

sj-docx-1-dst-10.1177_19322968251334633 – Supplemental material for Accuracy and Reliability of Intermittent Scanning and Real-Time Continuous Glucose Monitoring Systems in Diabetes Emergencies Supplemental material, sj-docx-1-dst-10.1177_19322968251334633 for Accuracy and Reliability of Intermittent Scanning and Real-Time Continuous Glucose Monitoring Systems in Diabetes Emergencies by Lukas van Baal, Lutz Heinemann, Johanna Reinold, Jill von Conta, Fin Hendrik Bahnsen, Jens Kleesiek, Dagmar Fuehrer and Susanne Tan in Journal of Diabetes Science and Technology
