# Supplementary material for: Accuracy and Reliability of Intermittent Scanning and Real-Time Continuous Glucose Monitoring Systems in Diabetes Emergencies
Source: J Diabetes Sci Technol. 2025 Apr 23:19322968251334633. Online ahead of print. doi: 10.1177/19322968251334633 (PMC12018364; doi:10.1177/19322968251334633)
Supplement: sj-docx-1-dst-10.1177_19322968251334633 – Supplemental material for Accuracy and Reliability of Intermittent Scanning and Real-Time Continuous Glucose Monitoring Systems in Diabetes Emergencies [file sj-docx-1-dst-10.1177_19322968251334633.docx]

**Supplementary Figure 1:** Department’s insulin infusion protocol


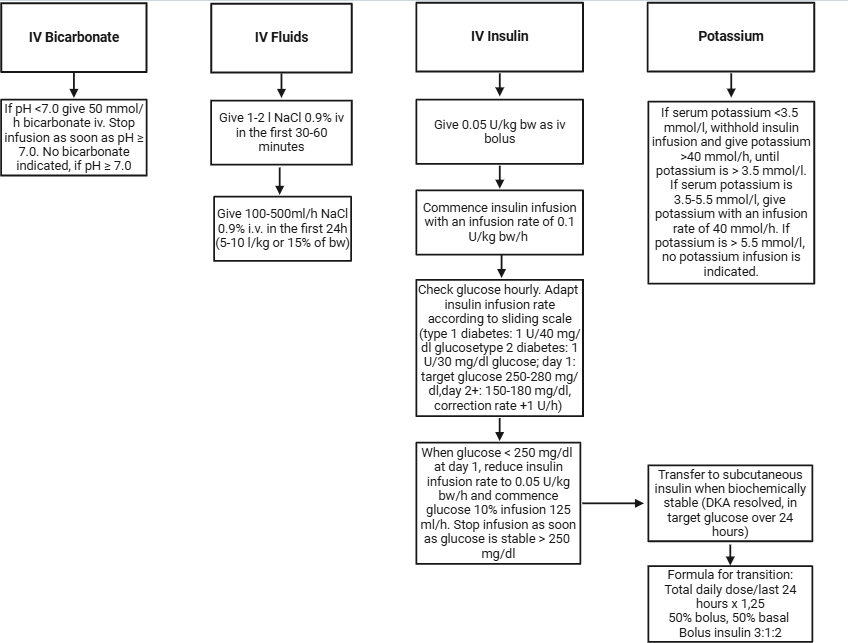

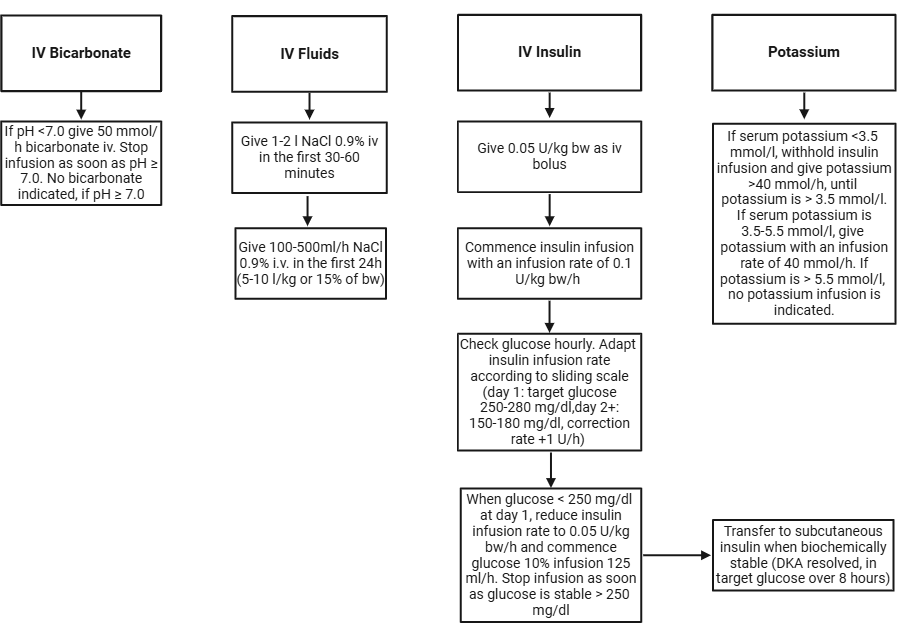


IV, intravenous; h, hour; NaCl, sodium chlorides; bw, body weight; U, unit; CGM, Continuous Glucose Monitoring; DKA, Diabetic Ketoacidosis

**Supplementary Figure 2:** Clarke Error Grid (CEG) and Bland Altman analysis for all FSL2-POC-G pairs (n = 521)

**
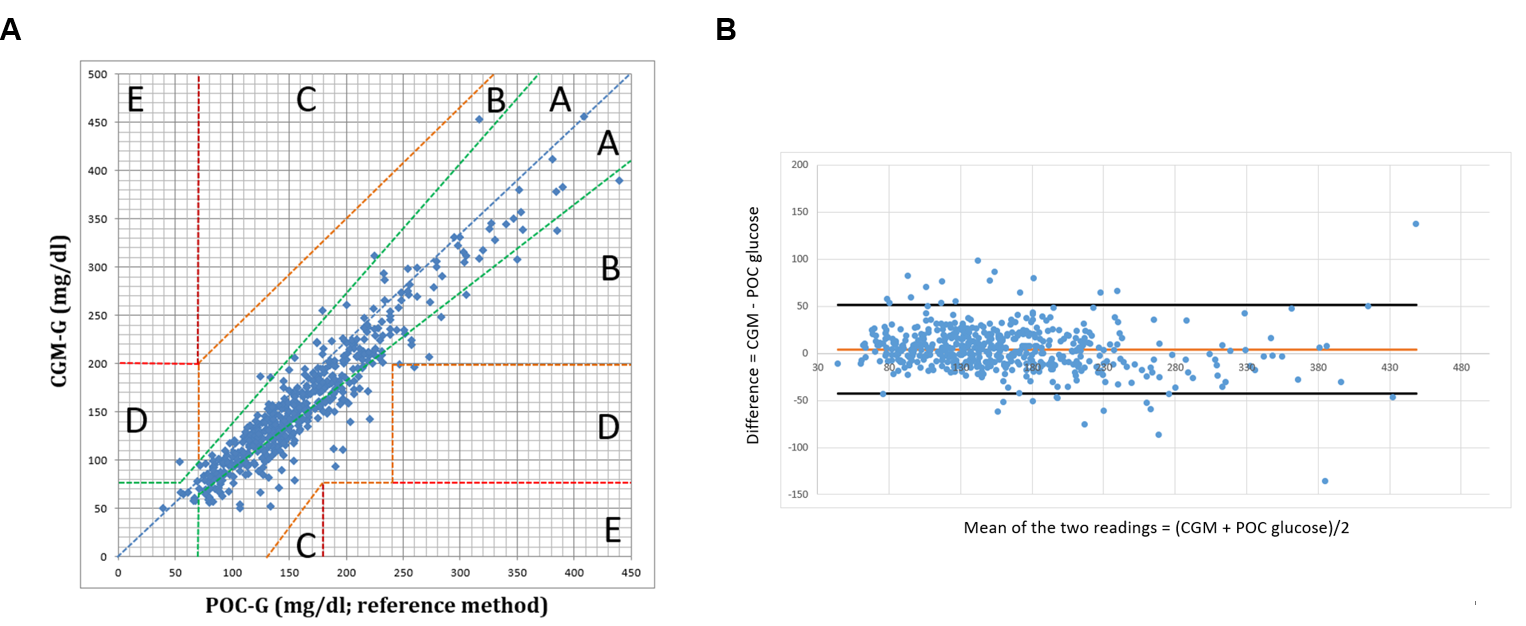
**

A: CEG for all FSL2-POC-G pairs (n = 521) within the measurement range of the CGM; B: Bland Altman plot of difference (CGM-1- POC) versus mean. This plot shows the difference between 521 matched CGM-G and POC-G data pairs vs. their mean. The mean difference is given by the solid line. Dashed lines note 2 standard deviation limits and provide an estimate of where 95% of the differences lie; CGM-G, Continuous Glucose Monitoring Glucose; POC-G, Point of Care Glucose; CGM, Continuous Glucose Monitoring; POC, Point of Care

**Supplementary Figure 3:** Clarke Error Grid (CEG) and Bland Altman analysis for all FSL3-POC--G pairs (n = 977)


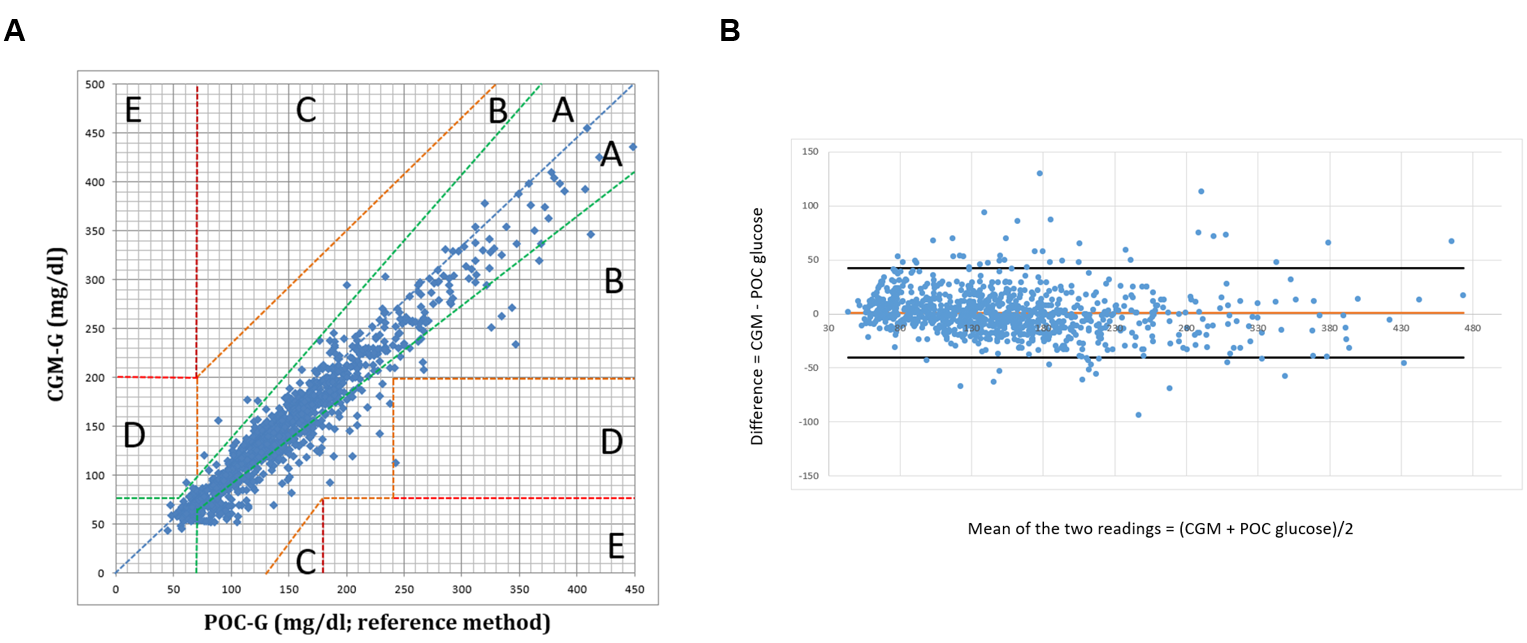


A: CEG for all FSL3-POC--G pairs (n = 977) within the measurement range of the CGM; B: Bland Altman plot of difference (CGM-2 - POC) versus mean. This plot shows the difference between 977 matched CGM-G and POC-G data pairs vs. their mean. The mean difference is given by the solid line. Dashed lines note 2 standard deviation limits and provide an estimate of where 95% of the differences lie; CGM-G, Continuous Glucose Monitoring Glucose; POC-G, Point of Care Glucose; CGM, Continuous Glucose Monitoring; POC, Point of Care


**Supplementary Table 1:** Accuracy of FSL2-G vs. POC-G according to FDA criteria

|  | within  +/- 0.3 mmol/l (5 mg/dl); +/- 5% | within  +/- 0.6 mmol/l (10 mg/dl);  +/- 10% | within  +/- 0.7 mmol/l (12 mg/dl);  +/- 12% | within  +/- 0.8 mmol/l (15 mg/dl);  +/- 15% | within  +/- 1.1 mmol/l (20 mg/dl);  +/- 20% | Total no. of pairs |
| --- | --- | --- | --- | --- | --- | --- |
| **Day 1** | 32.0% | 56.0% | 60.0% | 66.0% | 74.0% | 51 |
| **Day 2+** | 30.4% | 54.1% | 64.9% | 78.1% | 89.1% | 470 |
| **p** | 0.81 | 0.80 | 0.49 | 0.05 | **<0.01** | 521 |

Data are presented as percentage affected; p-value is provided for comparison of percentage affected on day 1 vs day 2+

**Supplementary Table 2:** Accuracy of FSL3-G vs. POC-G according to FDA criteria

|  | within  +/- 0.3 mmol/l (5 mg/dl); +/- 5% | within  +/- 0.6 mmol/l (10 mg/dl);  +/- 10% | within  +/- 0.7 mmol/l (12 mg/dl);  +/- 12% | within  +/- 0.8 mmol/l (15 mg/dl);  +/- 15% | within  +/- 1.1 mmol/l (20 mg/dl);  +/- 20% | Total no. of pairs |
| --- | --- | --- | --- | --- | --- | --- |
| **Day 1** | 34.9% | 60.2% | 63.0% | 69.4% | 75.9% | 108 |
| **Day 2+** | 35.6% | 59.8% | 66.9% | 77.0% | 87.6% | 869 |
| **p** | 0.89 | 0.94 | 0.42 | 0.08 | **<0.01** | 977 |

Data are presented as percentage affected; p-value is provided for comparison of percentage affected on day 1 vs. day 2+

**Supplementary Table 3:** Multiple regression analysis for potential confounders with MARD as dependent variable


|  | β | p-value |
| --- | --- | --- |
| BMI | -.06 | .49 |
| Age | -.01 | .96 |
| HbA1c | -.04 | .67 |
| Type of diabetes | .09 | .32 |
| Hemoglobin | .02 | .21 |
| Glomerular filtration rate | -.09 | .30 |
| Total bilirubin | .07 | .46 |
| Bicarbonate level at admission | .06 | .27 |

β, regression coefficient; BMI, Body Mass Index; HbA1c, Hemoglobin A1c
